# The Charlson Comorbidity Index Can Be Used Prospectively to Identify Patients Who Will Incur High Future Costs

**DOI:** 10.1371/journal.pone.0112479

**Published:** 2014-12-03

**Authors:** Mary Charlson, Martin T. Wells, Ralph Ullman, Fionnuala King, Celia Shmukler

**Affiliations:** 1 Center for Integrative Medicine, Weill Cornell Medical College, New York, NY, United States of America; 2 Department of Statistical Science, Cornell University, Ithaca, NY, United States of America; 3 1199SEIU Benefit and Pension Funds, New York, NY, United States of America; University of Milan, Italy

## Abstract

**Background:**

Reducing health care costs requires the ability to identify patients most likely to incur high costs. Our objective was to evaluate the ability of the Charlson comorbidity score to predict the individuals who would incur high costs in the subsequent year and to contrast its predictive ability with other commonly used predictors.

**Methods:**

We contrasted the prior year Charlson comorbidity index, costs, Diagnostic Cost Group (DCG) and hospitalization as predictors of subsequent year costs from claims data of fund that provides comprehensive health benefits to a large union of health care workers. Total costs in the subsequent year was the principal outcome.

**Results:**

Of the 181,764 predominantly Black and Latino beneficiaries, 70% were adults (mean age 45.7 years; 62% women). As the comorbidity index increased, total yearly costs increased significantly (P<.001). At lower comorbidity, the costs were similar across different chronic diseases. Using regression to predict total costs, top 5^th^ and 10^th^ percentile of costs, the comorbidity index, prior costs and DCG achieved almost identical explained variance in both adults and children.

**Conclusions and Relevance:**

The comorbidity index predicted health costs in the subsequent year, performing as well as prior cost and DCG in identifying those in the top 5% or 10%. The comorbidity index can be used prospectively to identify patients who are likely to incur high costs.

**Trial Registration:**

ClinicalTrials.gov NCT01761253

## Introduction

Health costs in the U.S. have been rising at an unsustainable pace [1], [2]. To have the best chance of reducing costs, interventions have to target those patients most likely to incur future high costs. The Congressional Budget Office evaluated three methods to identify individuals who would have future high costs, specifically, prior hospitalization, prior high costs and multiple chronic conditions; they found that multiple chronic conditions was the best predictor of sustained high costs [3]. These findings have been confirmed in other analyses [3]–[7]. Patients with multiple chronic diseases have increasingly become a critical focus for national efforts to reduce utilization and improve outcomes [3], [6], [8]–[11]. There is, however, there is no consensus about the criteria to define multiple chronic diseases and the definition varies considerably in different studies [12], [13]. DHHS has used a simple count of conditions for the definition of multiple chronic disease [14], citing a recent systematic review [12]. However, there is not agreement on what should be included in the count of conditions [13], [15]. The definitions of multiple chronic diseases recently used by the Agency of Healthcare Research and Quality Research Network varied considerably (i.e., more than 2 of 3–8 chronic diseases, diseases in the Chronic Conditions warehouse, or 28 conditions tracked by the Veterans Administration) [16], [17].

In this analysis, we operationally define multiple chronic diseases, using the Charlson comorbidity index [18]. Originally developed to predict survival, the index has been adapted for predicting costs [19]. The comorbidity index has been shown to predict costs in relation to many specific conditions (e.g., diabetes, cancer). The Comorbidity index, intrinsically a measure of aggregate chronic disease burden, has not been validated as a standalone predictor of future costs.

Our objective was to evaluate whether comorbidity, as assessed by the Charlson comorbidity index during one year, could be applied to a large population to identify those patients who would incur high health care costs in a subsequent year. The performance of the comorbidity index in predicting future costs was compared to prior year total costs, a usual benchmark; to the Diagnostic Costs Groups (DCG), one commonly used population-based risk adjustment strategy [20]; and to prior hospitalizations. Since mental health conditions commonly co-occur with chronic illness and are known to increase costs [21], [22], but are not included in the standard Charlson index (except for depression and dementia), their contribution to total costs was also evaluated.

## Methods

The study was reviewed and approved by the Weill Cornell Internal Review Board. Patient information was anonymized and de-identified prior to analysis; individual consent was not obtained.

### The population

The population is a union of health and hospital workers in the Northeast, whose self-insured trust fund administers comprehensive health benefits to the members, their spouses and children. The fund covers all medically necessary hospital, medical, maternity, behavioral health and pharmacy services, and maintains their own claims data repository. There were 226,157 individuals who were eligible for benefits for at least 11 months between January–December 2009; of those, 185,294 (86.2%) remained eligible for at least 11 months from January–December 2010. Overall 18.1% (n = 40,862) did not remain eligible for reasons such as retirement or dependent children reaching the maximum age of eligibility. This analysis focuses on the 181,764 beneficiaries who were consistently eligible for benefits over at least 22 months in 2009 and 2010, who also received DCG codes. (Sightlines DxCG Risk Solutions V 3.0, Verisk Health Inc)

### Demographic and clinical data

Beneficiary age and gender were available. Comorbidity was assessed through the Charlson comorbidity index [19]. Different weights are assigned for specific conditions and the weights are added to find the index for a specific patient (e.g., a patient with depression, COPD and lymphoma would have a weight of 4) (Table 1). Data on use of warfarin was not available.

**Table 1:** Different weights assigned for specific conditions in the comorbidity index.

Chronic disease	Weight	Chronic disease	Weight	Chronic disease	Weight
Cerebrovascular disease	1	Myocardial infarction	1	Skin ulcers/cellulitis	2
Congestive heart failure	1	Peripheral Vascular disease	1	Takes warfarin	1
COPD/Asthma	1	Rheumatic disease	1	Leukemia	2
Dementia	1	Ulcer disease	1	Lymphoma	2
Depression	1	Hemiplegia	2	Moderate/severe liver disease	3
Diabetes without end organ	1	Moderate/severe renal disease	2	Metastatic solid tumor	6
Hypertension	1	Diabetes with end organ damage	2	HIV/AIDS	6
Mild liver disease	1	Any tumor	2		

The comorbidity index was assessed from claims data for services provided between January and December 2009; each claim had at least one primary ICD-9 diagnosis code and up to three secondary codes. The comorbidity index was assessed using the Deyo strategy [23]. The comorbidity index for the first year was computed for all diagnoses recorded in all of the claims during the first year. 10.2% had no claims in 2009; of those, 63% were adults and 37% children. Among adults, of those who had no claims in 2009, 53% also had none in 2010. Among children, 43% of those who had no claims in 2009 also had none in 2010. Costs were zero for those without claims.

The prospective DCG model utilizes claims data and age, sex, diagnoses and their interactions to predict future costs; the 2009 prospective DCG was used to predict 2010 cost [24]. Mental health disorders encompassed the ICD-9 codes 290–316 other than depression (290.13, 296.20–296.36; 296.82; 298.0; 201.11; 301.12; 309.0–309.1 or 311) and dementia (290, 290.4–290.43, 331, 331.19, 331.2 or 331.82).

### Health care costs

Claims data was used to document the cost and utilization of services among these consistently eligible beneficiaries. The type and place of service were available. The prior year hospitalization was the actual number of hospitalizations. Total amount paid for all services including inpatient, outpatient, emergency room, laboratory tests, behavioral health and prescription drugs for January–December 2010 were evaluated.

### Data Analysis

To compare the costs for those with a given chronic disease versus those with that disease and other comorbid diseases, an adjusted comorbidity index was calculated by subtracting the Charlson comorbidity weight for that disease from the total comorbidity score. For example, patients with congestive heart failure (CHF) who have an adjusted index score of zero have only CHF, while those with an index of one or more have other illnesses as well.

Four predictors of subsequent yearly costs (i.e., prior year costs, prior year hospitalization, prior year comorbidity and prior year DCG) were compared using four different analytic approaches: a two part regression modeling strategy often used in econometric analysis; quantile regression to predict the upper 5% and 10% of the cost distribution; logistic regression to predict whether a specific individual would be in the upper 5% or 10% cost strata using positive predictive value; and receiver operating characteristic (ROC) analysis to evaluate sensitivity and specificity. Since cost data was skewed by both high cost patients and by those with zero costs, standard regression could not be used. Age, gender and mental health were controlled for in all regressions. There was no data on race/ethnicity in the claims; however, the beneficiaries are predominately Black and Latino. The data did not include the specific location where the beneficiaries received services.

#### A two part mean regression framework for modeling total health care cost

The first part of the two-part model is a binary outcome model that describes the distinction between non-users (zero cost) and users of services (non-zero cost), while the second part is a linear regression that describes the distribution of total health care cost for patients who used services (see File S1) [25]. As a diagnostic to assess the functional form for current and prior year (log) cost, we fit a nonparametric estimate of the relationship using a local polynomial smoother and found that the linear term was adequate. The higher order terms added little to the model fit statistics, consequently we went with the linear term.

*Quantile regression* was employed to assess the relationship between predictors and the upper tail of the cost distribution, controlling for age, gender and mental health diagnoses [26]; since it focuses on the upper tail, those with zero costs in the lower tail of the distribution do not heavily affect the estimates. The pseudo R^2^ is the measure of model fit [27]. The methodology for fitting a quantile regression model involves minimizing a weighted sum of absolute deviations. The pseudo-R^2^ in quantile regression is calculated as 1 – (sum of weighted deviations about estimated quantile)/(sum of weighted deviations about raw quantile) (see Koenker, R. 2005. Quantile Regression. New York: Cambridge University Press). We used this definition of pseudo-R^2^ for each of the models. The pseudo-R^2^ is analogous to the classical R^2^ = 1 – (error sum of squares)/(total sum of squares) from multiple regression.

*Positive predictive value* was assessed through logistic regression which was used to build a model that predicted whether an individual would fall into the top 5% and 10% highest predicted cost groups [28]. A 50% training set was used to generate 1000 new training sets by sampling uniformly and with replacement (the bootstrap samples); models were then fit using the bootstrap samples and combined for classification into the top 5% and 10% groups. This bagged bootstrap sampling was used to generate positive predictive values [29].

*Receiver operating characteristic* (ROC) analysis evaluates the sensitivity and specificity at various threshold settings of prior year costs, prior year comorbidity, prior year hospitalizations and prior year DCG to predict membership in the top 5% and 10% cost groups [30]. The area under the curve (AUC) used to compare classifiers.

## Results

Overall, 69.3% of the predominantly Black and Latino beneficiaries were adults (mean age 45.7±12.3 years; 62.3% women), while 30.7% were children less than 18 years of age (3.9% less than one year; 21.3%, 1–6 years; 34.0%, 6–11 years; and 40.8%, 12–17 years). With regard to the adults, 75.3% were union members; 20.5%, their spouses; and 4.2%, their covered adult children (mean age 19.5±1.2 years). 99.2% of the beneficiaries live in the New York tristate area where they receive care through many different physicians, practices and hospitals.

Table 2 shows the beneficiaries, adults or children, according to the prior year comorbidity index and their subsequent adjusted yearly costs. (Table S1 in File S1 shows the unadjusted costs; it also shows results were monotonic and did not depend on specific groupings).Overall, 89.0% of the costs were incurred by adults and 11.0% by children. Adults had a mean yearly cost of $4,371 and children, a mean cost of $1,324 (median costs $1,879 and $533, respectively).

**Table 2:** Beneficiaries and 2010 health care costs according to the prior year Charlson comorbidity index.

*Adults*					
Comorbidity beneficiaries according to comorbidity and 2010 cost. Comorbidity index	Beneficiaries	% beneficiaries Percen%memmb Members	Total cost	% costs	Cost per person person
0–1	88,446	70.2%	$271,440,774	45.6%	$3,303
2–3	25,907	20.6%	$171,245,270	28.8%	$6,168
4	5,415	4.3%	$48,442,590	8.1%	$8,248
5–7	4,874	3.9%	$66,817,666	11.2%	$12,946
≥8	1,322	1.1%	$37,454,904	6.3%	$27,416

Children					
Comorbidity beneficiaries according to comorbidity and 2010 cost. Comorbidity index	Beneficiaries	% beneficiaries Percen%memmb Members	Total cost	% costs	Cost per person
0–1	53,919	96.6%	$66,913,479	90.3%	$1,247
2–3	1,754	3.1%	$5,842,574	7.9%	$3,202
4	63	0.1%	$604,737	0.8%	$9,371
5–7	51	0.1%	$616,182	0.8%	$12,063
≥8	13	0.0%	$135,018	0.2%	$10,814

*Adults and Children*					
Comorbidity beneficiaries according to comorbidity and 2010 cost. Comorbidity index	Beneficiaries	% beneficiaries Percen%memmb Members	Total cost	% costs	Cost per person
0–1	142,365	78.3%	$338,401,605	50.5%	$2,662
2–3	27,661	15.2%	$177,085,722	26.5%	$5,508
4	5,478	3.0%	$49,044,534	7.3%	$7,709
5–7	4,925	2.7%	$67,433,100	10.1%	$12,377
≥8	1,335	0.7%	$37,576,245	5.6%	$26,707

Yearly cost per person adjusted for age, gender, major mental health diagnoses, and zip code of residence.

The total health care costs in 2010 were $670 million. Beneficiaries with a comorbidity index ≥4 constituted only 6.5% of the population, but accounted for $154 million, or 23.0% of total 2010 costs. As expected, adults and children differed significantly in comorbidity (p<.001); only 0.2% of children had a comorbidity index ≥4 in comparison to 9.2% of the adults.

### Comorbidity and health care costs

**Figure 1:**
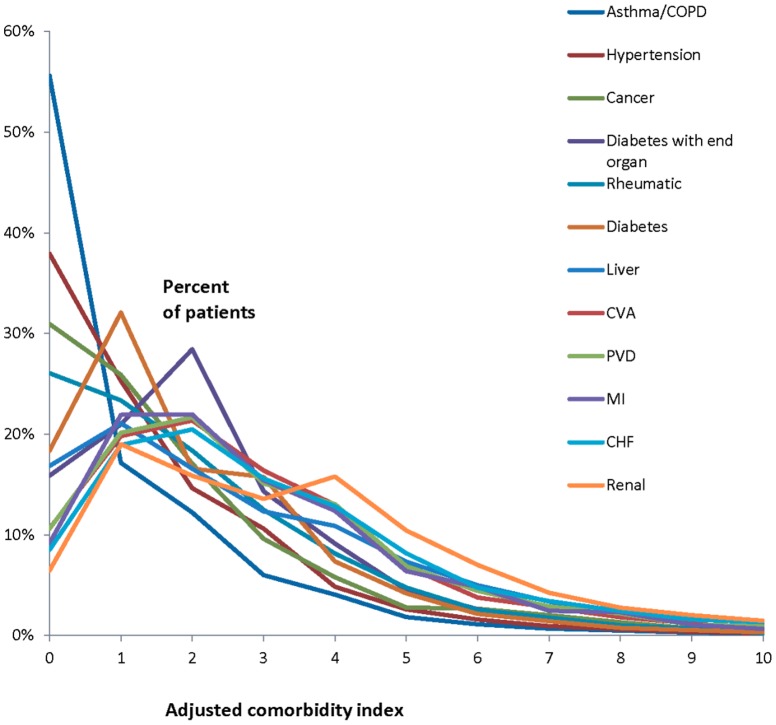
The y axis is the proportion of patients with a given chronic disease according to the adjusted comorbidity index. The x-axis is the adjusted comorbidity index found by subtracting the weight of each disease from the patient's comorbidity index *for those patients with the stated disease*. Thus, a patient with an adjusted comorbidity index of 0 has only that chronic disease.

Figure 1 shows the distribution of beneficiaries according to the adjusted comorbidity index for the specific chronic diseases. Those with an adjusted comorbidity index of zero have only the specific chronic disease. Regardless of specific chronic disease, most patients had low comorbidity. Only a small proportion of patients with any specific chronic disease had high comorbidity. (See Table S2 in File S1 for numbers of patients in each category.)

**Figure 2:**
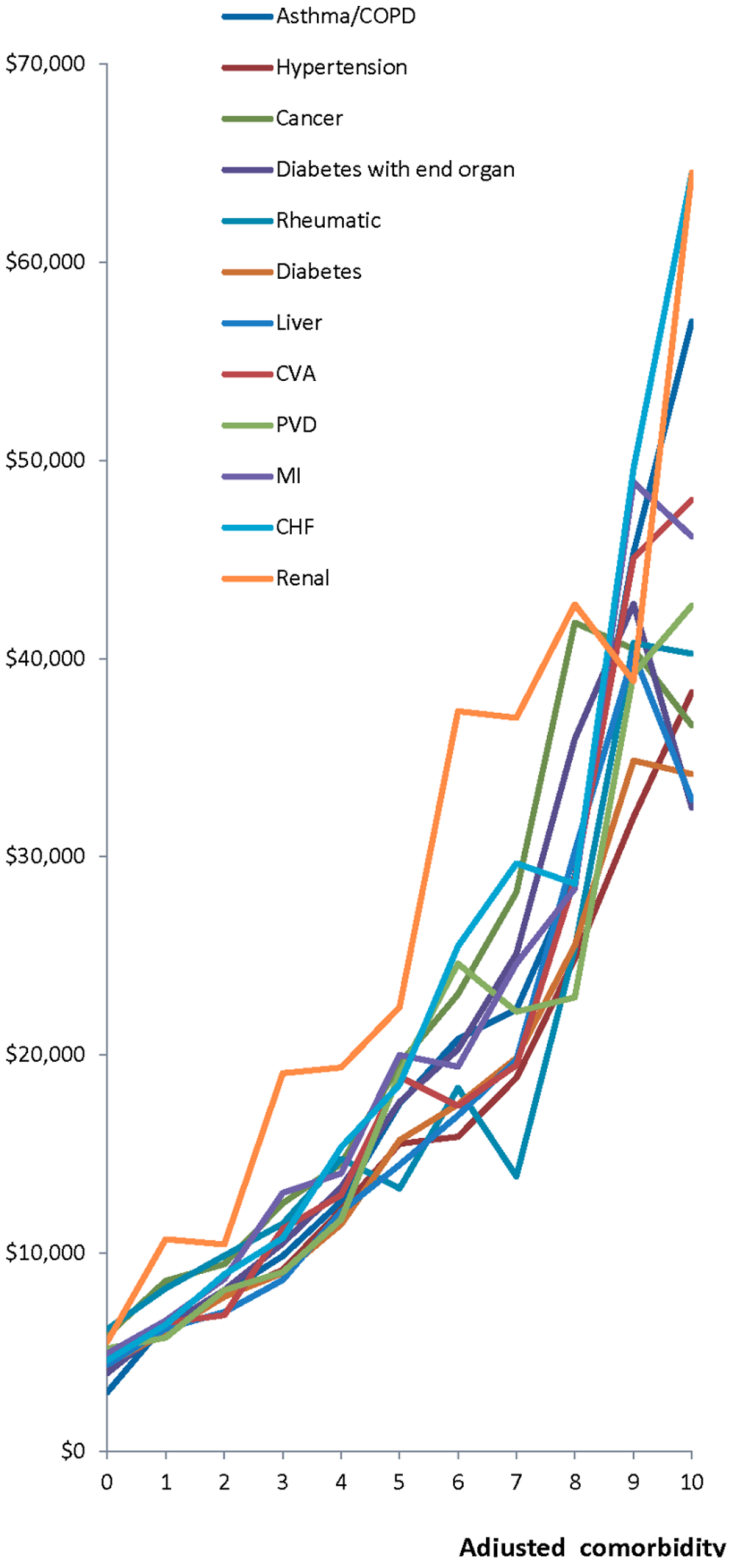
The y-axis is total costs, that is, the total yearly costs for patients with that disease according to the comorbidity level. The x-axis is the adjusted comorbidity index found by subtracting the weight of each disease from the patient's comorbidity index *for those patients with the stated disease*. Thus, a patient with an adjusted comorbidity index of 0 has only that chronic disease.

Figure 2 shows the adjusted comorbidity index and total costs. As the index increased, total yearly cost increased significantly, regardless of the specific chronic disease (P<.001). At lower comorbidity, the costs did not differ significantly for different chronic diseases.

### Comorbidity and hospitalization

Overall 6.4% of adults and 2.0% of children were hospitalized. Table 3 shows hospitalization rates according to comorbidity. As comorbidity index increased, the proportion of beneficiaries hospitalized once, twice or three or more times increased significantly (P<.001). Patients with higher comorbidity had more hospitalizations (p<.001) and more repeated hospitalizations (p<.001). Table 3 also shows the costs for beneficiaries according to the comorbidity index and the number of hospitalizations. Costs steadily rose with increasing comorbidity index (p<.001) and increasing hospitalizations (p<.001).

**Table 3:** Percent of beneficiaries according to 2010 hospitalizations and average 2010 yearly costs according to prior year comorbidity index.

	Hospitalizations							
Comorbidity	None		One		Two		Three or more	
Adults Adults	Percent	Cost	Percent	Cost	Percent	Cost	Percent	Cost
0–1	95.0%	$ 2,190	4.2%	$15,536	0.6%	$36,404	0.2%	$ 59,105
2–3	91.7%	$ 4,704	6.5%	$20,400	1.2%	$42,413	0.5%	$ 88,281
4	90.0%	$ 6,492	7.5%	$22,584	1.8%	$52,961	0.7%	$ 61,981
5–7	85.6%	$ 8,999	9.9%	$26,737	2.6%	$52,027	1.9%	$105,352
≥8	76.0%	$15,928	13.6%	$42,987	4.4%	$64,817	6.0%	$126,995
**Children**								
0–1	98.1%	$ 955	1.6%	$11,534	0.2%	$30,836	0.1%	$ 56,690
2–3	95.0%	$2,260	3.9%	$15,891	0.9%	$ 58,071	0.2%	$ 34,700
≥4	88.9%.	$5,684	5.5%	$10,182	0.8%	$ 18,558	4.7%	$79,314

**The numbers of patients in each comorbidity group are shown on** **Table 1** **.**

Costs adjusted for age, gender, major mental health diagnoses, and zip code of residence.

### Comorbidity and mental/behavioral health

The only mental or behavioral health conditions in the comorbidity index are depression and dementia. 0.9% of the beneficiaries had dementia, and only 2.9% of adults (average age 45.2) and 1.0% of children (average age 13.8) had a diagnosis of depression. In total, 4.9% of adults (average age 45.2) and 1.0% of children (average age 11.3 years) had another mental health diagnosis. Overall, 5.9% of beneficiaries who had a mental health diagnosis (other than depression or dementia) accounted for 11.0% of the costs (p<.001). The frequency of a mental health diagnosis increased with increasing levels of comorbidity. For those with a comorbidity index of 0–1, 5% had a mental health diagnosis; comorbidity of 2–3, 8.8%; comorbidity of 4, 9.4%; comorbidity 5–7, 10.2% and those ≥8, 14.1%. At each level of comorbidity, those with mental health diagnoses had significantly increased costs (p<.001).

### Predictive modeling

Table 4 shows predictions of the 2010 total expenditure from regressions that included either prior year costs, prior year comorbidity index, prior year hospitalization or the DCG prospective risk score. Total costs in 2009 were $4,256 for adults and $1,410 for children, very similar to those in 2010. Overall 5.6% of adults and 2.3% of children were hospitalized in 2009, also very similar to 2010; most of the adults and children were hospitalized only once. The mean prospective DCG score in 2009 was 1.6±2.2.

**Table 4:** Regression models evaluating prior year (2009) predictors of subsequent year (2010) costs.

	Adults			Children			Both		
Independent variables	t	p	R^2^	t	p	R^2^	t	p	R^2^
Prior year costs	175.6	<.01	.31	97.0	<.01	.20	205.6	<.01	.37
Comorbidity index	104.0	<.01	.20	17.2	<.01	.17	114.3	<.01	.25
Prior year hospitalization	28.1	<.01	.11	16.3	<.01	.07	34.9	<.01	.18
Prospective Risk Score	125.8	<.01	.20	51.2	<.01	.07	139.1	<.01	.26

Each model had only the single independent variable in the first column, controlling for age, gender and mental health diagnosis.

t indicates the strength of the association and the p value the statistical significance. R^2^ is the explained variance, that is, the extent to which the prior year variables predict subsequent year costs; the higher the R^2^, the greater the explanatory or predictive power.

Overall, 9.7% of beneficiaries had zero costs in 2010. Those with lower comorbidity (p<.001), without a mental health diagnosis (p<.001), men (p<.001), and those with zero costs in 2009 (p<.001) were more likely to have zero costs in 2010. In adults, prior year costs explained 31% of the variance, while the comorbidity index and prospective DCG each explained 20%; prior hospitalization explained only 11%. In children, prior costs explained 20% of the variance, and the comorbidity index explained 17% of the variance, while prospective DCG and prior hospitalization explained 7%. Each model in Table 4 is controlled for age, gender and mental health diagnosis. Random effects for zip code and fixed effects for employer type were not significant and were omitted from the regression models in Table 4.

Table 5 shows the results of quantile regressions for predicting the top 5^th^ and 10^th^ percentile of costs for both adults and children. Prior year costs, prior year comorbidity, prior year DCG, and prior year hospitalizations were all evaluated as predictors of upper 5% and upper 10% of subsequent (2010) costs in separate models controlling for age, gender and mental health diagnosis. In adults, the comorbidity index was equivalent to DCG and prior cost in predicting the top 5% and 10% of cost, while prior hospitalization had much lower ability to identify such patients. In children, the comorbidity index, prior costs, and DCG also had similar predictive ability for the top 5% and 10% of costs. Table S3 in File S1 shows the percent of patients in each prior year comorbidity rank who fell into the upper 0.5%, 1.0%, 2.0%, 5.0%, and 10% of subsequent year costs. Since there is a monotonic gradient of increasing costs with increasing comorbidity, the findings are not contingent on a specific cutoff.

**Table 5:** Predictors of the adults and children who would have the top 5% and 10% of subsequent (2010) costs using quantile regression.

	Top 5% of costs			Top 10% of costs		
Adults	t	p	Pseudo R^2^	t	p	Pseudo R^2^
Prior year costs	53.6	<.01	.13	90.7	<.01	.14
Prior year comorbidity	42.7	<.01	.12	62.9	<.01	.13
Prior year DCG Score	76.1	<.01	.12	120.7	<.01	.12
Prior year hospitalizations	28.9	<.01	.04	37.6	<.01	.04
**Children**						
Prior year costs	35.2	<.01	.11	52.7	<.01	.12
Prior year comorbidity	22.7	<.01	.10	33.6	<.01	.11
Prior year DCG Score	89.8	<.01	.10	126.1	<.01	.09
Prior year hospitalizations	25.9	<.01	.03	35.9	<.01	.03

Each model had only a single independent variable controlling for age, gender and mental health diagnosis.

t indicates the strength of the association and the p value the statistical significance. R^2^ is the explained variance, that is, the extent to which the prior year variables predict subsequent year costs; the higher the R^2^, the greater the explanatory or predictive power.

Another approach to classification assessed how likely it was for an individual to be in the top 5% and 10% of 2010 costs based on prior year characteristics. Table S4 in File S1 shows the positive predictive values for predicting 2010 costs from bootstrapping using prior year costs, prior year comorbidity, prior year hospitalizations and prior year DCG to predicting membership in the top 5% and 10% of 2010 cost, controlling for age, gender and mental health diagnosis. Prior year comorbidity had a slightly higher positive predictive value for identifying those who would become a member of the top 5% and 10% subsequent year cost than hospitalization and both are better than prior year costs and prior year DCG score. The positive predictive values are relatively stable across the top 5% and 10% cost groups. With respect to the ROC analysis, the comorbidity index achieved almost identical area under the curve as prospective DCG and prior cost in predicting the top 5% and 10% of subsequent costs, these three measures are slightly better than prior years hospitalization. The small difference between the findings for positive predictive value and area under the curve is that the positive predictive value depends on the prevalence of high costs as well as on sensitivity and specificity. Each model in Table 5 is controlled for age, gender and mental health diagnosis. Again, in this set of regression models random effects for zip code and fixed effects for employer type were not significant and were omitted from the regression models in Table 5.

## Discussion

In this study, multiple chronic disease was operationally defined using the Charlson comorbidity index. Cited in more than 9,500 publications, the comorbidity index is the most extensively validated measure of the prognostic impact of multiple chronic illnesses. The prior year comorbidity index was compared to prior year cost, prior year DCG and prior year hospitalization as a predictor of subsequent year costs using two part regression and quantile regression as well as several classification approaches.

With two part regression the comorbidity index explained 20% of the overall variance in adults vs. 31% for the benchmark prior year costs, 20% for DCG and 11% for prior hospitalizations. The comorbidity index did almost as well as prior cost in children (17% vs. 20%), better than DCG (7%), or hospitalizations (7%). A similar analysis evaluating predictors of costs among VA patients enrolled in an ambulatory care quality improvement trial found that the adjusted R^2^ for prior year costs was 4.9%; DCG, 7.2%, and the comorbidity index, 3.6%; however, the explained variance was low because costs were estimated base on units of service (visits, hospitalizations) and not on actual expenditures [31]. In the quantile regression analysis, for both adults and children, the comorbidity index did slightly less well than prior year costs and as well or better than DCG in predicting the crucially important highest-cost patients, represented as the upper 5^th^ and 10^th^ percentile. One study that used the Medical Expenditure Panel Survey data to predict the top 10% of costs found that the pseudo R^2^ for DCG was identical to our study, 20% [32]. A recent evaluation of adults in the upper 10% of costs who receive care from the Mayo Employee and Community Health practice found that CMS-HCC and Charlson comorbidity index had identical explanatory power as assessed by the C statistic [33]. In both of these analyses, the amount of explained variation, while relatively low, is typical for models predicting yearly costs including those currently used by CMS [20].

Two classification approaches positive predictive value and ROC analysis were also used to classify patients according to their likely membership in high cost groups. Prior year comorbidity had a slightly higher positive predictive value than prior hospitalization and both were better than prior costs and prior DCG score. With respect to the ROC analysis, the comorbidity index achieved almost identical area under the curve as prospective DCG and prior cost in predicting the top 5% and 10% of subsequent costs.

### Limitations

These results were obtained from analysis of a specific population and cannot be directly compared to the explanatory power of other published cost models, in part because any analysis of costs, including this one, encompass differentials in provider pricing. However, contrasting the different models in this population provides a context for evaluating the predictive ability of prior comorbidity, prior cost, prospective DCG and prior hospitalizations to predict subsequent costs in the same population. The comorbidity index could not be calculated for the 10% who did not have claims in the first year.

In addition, the comorbidity index has not been independently validated in children, although it has been used in some pediatric studies [34]–[36]. The comorbidity index does not capture a variety of conditions unique to pediatrics. Although children who have illnesses that in the index do accumulate higher costs, further modification would be required to adapt it to the chronic conditions unique to children. It should also be noted that supply side factors were not considered in this analysis.

### The importance of the findings

To be successful in reducing health care expenditures, interventions have to identify the patients who are most likely to have sustained high costs. If not targeted precisely toward such patients, interventions will cost more than they will save. This was the experience with disease management interventions [37]–[40]. Studies have shown that both prior costs and prior hospitalization overestimate subsequent health care utilization and neither reliably identifies those who have stable high costs from year to year [3], [6]. Likely as a result, intervention programs that have targeted beneficiaries using these definitions have not demonstrated savings [3], [41]. Thus, to date, most interventions have not been successful in reducing costs in patients with multiple chronic diseases. Our hypothesis is that they did not efficiently target the patients at highest risk of high future costs.

Focused on adults, a comorbidity index ≥5 identifies 5% of patients who will have 17.5% of costs; or comorbidity index ≥4 identifies 9.3% of patients who will incur 25.6% of costs. A recent study using the VA criteria found that 32% of patients had 65% of costs [42]. The issue is the tradeoffs between the percent of patients and the percent of possible reduction in future costs.

This study used the comorbidity index calculated for patients from claims data from the prior year to predict subsequent year costs and contrasted it with commonly used predictors of cost including prior hospitalization, prior costs, and DCG. Prior costs and prior DCG usually require 12 months of claims data, which generally creates a lag time of 6–18 months [43]. Accordingly, such data cannot be used to identify risk for high costs among patients for whom health plans or employers have no prior utilization data, including those who may gain insurance under the Affordable Care Act. Prior hospitalization might be ascertainable at the time of enrollment but clearly affords less explanatory power.

Importantly, the Charlson comorbidity index can also be documented prospectively by a short questionnaire as well as through claims or chart data. For more than 20 years, we and others have ascertained the comorbidity index in less than 10 minutes through interview in person or by telephone [44], [45].

## Conclusions

Among 181,764 beneficiaries, the Charlson comorbidity index from a prior year predicted high health costs in the subsequent year. The index performed as well as prior cost or DCG in identifying those who would have higher costs, and those who would fall in the top 5% or 10% of costs in both adults and children. The comorbidity index also predicted subsequent hospitalization and repeated hospitalization, the largest component of costs. The comorbidity index provides a reproducible and prospectively applicable method of identifying patients at high risk of incurring high subsequent costs. As a reproducible, clinically validated and widely used method that has commensurate face validity for clinicians, the Charlson comorbidity index shows where the cost curve bends [46].

## Supporting Information

Data S1 (ZIP)

File S1 **Contains the description of the two-part regression.** **Table S1**, Yearly costs for adults and children according to each rank of the comorbidity index. **Table S2**, Number of patients with specific chronic diseases according to the adjusted comorbidity score. **Table S3**, Percent of patients falling into the upper 0.5%, 1%, 2%, 5% and 10% of 2010 costs based on 2009 comorbidity index. **Table S4**, Classification of individual's likelihood of membership in the top 5% or top 10% of subsequent costs according to different prior year characteristics.
(PDF)
