# Supplementary material for: The Charlson Comorbidity Index Can Be Used Prospectively to Identify Patients Who Will Incur High Future Costs
Source: PLoS One. 2014 Dec 3;9(12):e112479. doi: 10.1371/journal.pone.0112479 (PMC4254512; doi:10.1371/journal.pone.0112479)
Supplement: File S1 — Contains the description of the two-part regression. Table S1, Yearly costs for adults and children according to each rank of the comorbidity index. Table S2, Number of patients with specific chronic diseases according to the adjusted comorbidity score. Table S3, Percent of patients falling into the upper 0.5%, 1%, 2%, 5% and 10% of 2010 costs based on 2009 comorbidity index. Table S4, Classification of individual's likelihood of membership in the top 5% or top 10% of subsequent costs according to different prior year characteristics. (PDF) [file pone.0112479.s002.pdf]

Appendix

Two part regression: Classical mean-based regression models examine the center of the distribution so large number of zeros in cost data may skew the analysis; to avoid this, the data was analyzed using a two-part mean regression model which has had widespread use.[21 45-48] In a two-part model, explanatory variables often play different roles in the two parts of the model. Since the nonzero expenditure data are always positive and heavily skewed to the right, they are modeled with a lognormal distribution. Let Z_i denote the observed total cost for the ith subject and $\boldsymbol{X}_i$ represent a vector of subject specific characteristics. With inflated zero values, the observed costs are assumed to represent realizations of random variables that have probability distributions describable by a mixture of a point mass at zero and a continuous distribution. That is, $Z_i = I[Y_{1i} > 0] \times exp\{Y_{2i}\}$, where Y_{1i} and Y_{2i} represent two latent random variables, $I[A]$ denotes the indicator of the event *A (= 1 if A occurs and = 0 otherwise)*, and $exp\{ . \}$ is the exponential function.[49] Intuitively, Y_{1i} regulates when zero costs occurs and Y_{2i} is the logarithm of non-zero costs. Zhang et al.[50] extend the two-part structure in Duan[21] to model the distributions of the two latent random variables Y_{1i} and Y_{2i}. This model is at the individual level and adjusts for individual level characteristics. Specifically,

$$Z_i = I[Y_{1i} > 0] \times exp\{Y_{2i}\},$$

$$\text{Model 1: } Y_{1i} = \alpha_0 + \alpha_1 \boldsymbol{X}_{1i} + \varepsilon_{1i} \quad \varepsilon_{1i} \sim N(0, 1) \qquad (1)$$

$$\text{Model 2: } Y_{2i} = \beta_0 + \beta_1 \boldsymbol{X}_{2i} + \varepsilon_{2i} \quad \varepsilon_{2i} \sim N(0, \sigma_2^2). \qquad (2)$$

In these models, the covariates $\boldsymbol{X}_{1i}$ and $\boldsymbol{X}_{2i}$ can be different subsets of the complete set of observed characteristics, $\boldsymbol{X}_i$ and the variance of ε_{1i} is set to one for identifiability. A consequence of Model 2 is that the actual nonzero costs are specified as a log-normally distributed random variable. An important feature of a log-normally distributed random variable model is that the variance is exponentially increasing in the levels of the $\boldsymbol{X}_{2i}$.[50] Estimation for the models defined by (1) and (2) is carried out using maximum likelihood estimation standard

errors are computed using a parametric bootstrap procedure.   The coefficient of variation are computed from the estimates derived from the log transformed continuous part of the model.

The beta coefficients are from the log cost regression for the main effect of prior year costs, prior year comorbidity, prior year hospitalizations and prior year DCG in Equation 2.

Supporting Table S1 Yearly costs for adults and children according to each rank of the comorbidity index

	All beneficiaries mean yearly cost	Number of beneficiaries	Adults mean yearly Costs	Number of adults	Children mean yearly costs	Number of children
0	$1,883	106,175	$2,493	60,570	$1,072	45,605
1	$3,824	36,190	$4,318	27,876	$2,167	8,314
2	$5,778	18,502	$6,012	17,120	$2,869	1,382
3	$7,662	9,159	$7,773	8,787	$5,045	372
4	$8,953	5,478	$8,945	5,415	$9,599	63
5	$11,831	2,475	$11,802	2,460	$16,597	15
6	$14,939	1,590	$14,985	1,565	$12,096	25
7	$16,738	860	$16,878	849	$5,892	11
≥8	$28,147	1,335	$28,322	1,322	$10,385	13

Supporting Table S2. Number of patients with specific chronic diseases according to the adjusted comorbidity score

	Adjusted comorbidity index											
**Chronic illness**	**0**	**1**	**2**	**3**	**4**	**5**	**6**	**7**	**8**	**9**	**10**	**≥11**
Asthma/COPD	12,429	3,833	2,731	1,341	907	411	249	152	113	68	49	65
Hypertension	16,020	10,683	6,182	4,486	2,052	1,100	676	396	260	157	100	126
Cancer	2,867	2,399	1,582	890	538	259	246	187	123	65	41	65
Diabetes with end organ damage	1,158	1530	2,073	1,046	666	330	192	112	64	47	32	43
Rheumatic	821	736	579	394	257	150	81	56	32	22	8	15
Diabetes	3,933	6,878	3,556	3,376	1,577	900	462	305	166	116	72	95
Liver	314	393	309	230	203	137	93	63	36	30	26	30
CVA	276	514	554	425	337	178	98	73	47	31	17	43
PVD	403	766	824	575	496	262	169	110	83	41	36	37
MI	194	463	464	326	262	135	102	52	49	23	14	29
CHF	192	426	461	351	289	184	107	77	55	36	30	43
Renal	70	206	172	147	171	113	76	46	30	22	16	14

For example, patients with any given chronic disease (i.e., congestive heart failure) who have an adjusted index score of zero have only that disease, while those with an index of one or more have other illnesses as well.

Supporting Table S3 Percent of patients falling into the upper 0.5%, 1%, 2%, 5%, and 10% of 2010 costs based on 2009 comorbidity index

Comorbidity	Upper 0.5%	Upper 1.0%	Upper 2.0%	Upper 5.0%	Upper 10.0%
0	0.2%	0.3%	0.7%	4.1%	4.8%
1	0.3%	0.8%	1.8%	10.0%	11.7%
2	0.8%	1.6%	3.1%	16.9%	20.0%
3	1.4%	2.6%	4.8%	23.8%	28.6%
4	1.2%	2.9%	5.8%	31.0%	36.9%
5	2.1%	4.4%	9.0%	40.6%	49.6%
6	3.0%	6.0%	12.9%	52.8%	65.7%
7	4.5%	6.8%	16.7%	53.8%	70.5%
≥8	12.3%	17.8%	30.2%	64.7%	94.9%

Supporting Table S4 Classification of individual's likelihood of membership in the top 5% or top 10% of subsequent costs according to different prior year characteristics

	Positive predictive value		Area under the ROC curve	
	**Top 5% of costs**	**Top 10% of costs**	**Top 5% of costs**	**Top 10% of costs**
Prior year costs	51.1%	55.7%	.66	.68
Prior year comorbidity	62.1%	62.7%	.65	.65
Prior year DCG Score	52.0%	53.1%	.66	.67
Prior year hospitalizations	58.0%	61.7%	.56	.55

Each model had only a single independent variable controlling for age, gender and mental health diagnosis.
